# MiR-135-5p inhibits TGF-β-induced epithelial-mesenchymal transition and metastasis by targeting SMAD3 in breast cancer

**DOI:** 10.7150/jca.47083

**Published:** 2020-09-09

**Authors:** Wen Yang, Wen Feng, Fenglei Wu, Yuan Gao, Qian Sun, Nan Hu, Wei Lu, Jun Zhou

**Affiliations:** 1 Department of Obstetrics and Gynecology, The First People's Hospital of Lianyungang, Jiangsu 222061, P.R. China.; 2 Department of Oncology, The First People's Hospital of Lianyungang, Jiangsu 222061, P.R. China.; 3 Department of Breast surgery, The First People's Hospital of Lianyungang, Jiangsu 222061, P.R. China.

**Keywords:** breast cancer, microRNA-135-5p, SMAD3, epithelial-to-mesenchymal transition, metastasis.

## Abstract

Breast cancer (BC) is the most frequently diagnosed malignant tumors and the leading cause of death due to cancer in women around the world. A growing body of studies have documented that microRNA (miR)-135-5p is associated with the development and progression of BC. Considering that sekelsky mothers against dpp3 (SMAD3) plays a crucial role in transforming growth factor (TGF)-β/SMAD pathway and epithelial-mesenchymal transition (EMT) process, it is critical to elucidate the crosstalk and underlying regulatory mechanisms between miR-135-5p and SMAD3 in controlling TGF-β-mediated EMT in BC metastasis. Our results revealed a reciprocal expression pattern between miR-135-5p and SMAD3 mRNA in BC tissues and cell lines. Moreover, miR-135-5p was decreased in BC tissues compared to adjacent breast tissues; more interesting, miR-135-5p mRNA levels (Tumor/Normal, T/N) was further decreased in BC patients with lymph node metastasis, while SMAD3 mRNA levels were increased. Gain- and loss-of-function assays indicated that overexpression of miR-135-5p inhibited TGF-β-mediated EMT and BC metastasis *in vitro* and* in vivo*. Furthermore, knockdown of SMAD3 produced a consistent phenotype of miR-135-5p overexpression in breast cancer cells. Mechanistically, SMAD3, a pivotal transcriptional modulator of TGF-β/SMAD pathway, for the first time, was analyzed and identified as a target gene of miR-135-5p by bioinformatic algorithms and dual-luciferase reporter assays. Taken together, we clarified that miR-135-5p suppressed TGF-β-mediated EMT and BC metastasis by negatively regulating SMAD3 and TGF-β/SMAD signaling. Our findings supported that miR-135-5p may serve as a tumor suppressor, and be a valuable diagnostic biomarker for the treatment of BC.

## Introduction

Breast cancer (BC) is one of the most frequently diagnosed cancer, which has the highest cancer-associated mortality rate in women worldwide 1,2. Despite advances in surgery, chemotherapy and radiation therapy in treating BC, patients with BC yet exhibits poor prognosis, which is mainly caused by therapy resistance and metastasis 3,4. Therefore, it is important to clarify the molecular mechanisms underlying BC metastasis, which may provide better therapeutic strategies for BC.

miRs are a kind of endogenous small non-coding RNAs (~22 nucleotides in length) that play crucial roles in negatively regulating gene expression mainly by triggering mRNA degradation or translation repression by binding to the 3′-untranslated region (UTR) of target genes 5,6. Emerging evidence has documented that dysregulation of some miRNAs are tightly linked with the development and progression of BC 7,8. It has been documented that miR-135 majorly serves as a tumor suppressor in various types of human cancer, including BC 9-11. Jiang *et al* reported that miR-135-5p was downregulated and affected the proliferation and EMT of BC cells by controlling the Wnt/β-catenin pathway 11. Moreover, Yang *et al* demonstrated that miR-135-5p acts as a tumor promoter in pancreatic cells adaptation to metabolic stress 12. This contradiction is mainly due to tumor heterogeneity in the microenvironment or target genes. However, whether and how miR-135-5p regulates TGF-β-mediated EMT and BC metastasis has not fully understood yet.

It is well studied that epithelial-mesenchymal transition (EMT) is a key event in tumor metastasis, especially in the transformation of early-stage tumors to late-stage tumors 13,14. Specifically, cells undergoing EMT lose epithelial characteristics, including specialized cell-cell adhesion and cell polarity, then acquire enhanced migratory and invasive ability 15. During EMT, the expression of mesenchymal marker, such as Vimentin and Snail, is significantly increased, while that of epithelial cell marker E-cadherin is markedly decreased 16,17. As an indispensable transcriptional modulator of TGF-β/SMAD pathway, SMAD3, deeply involved in the pathogenesis of carcinogenesis and metastasis. Recent studies have revealed that aberrant activation of TGF-β/SMAD pathway trigger EMT and metastasis is involved in the development and progression of human cancers 18-20. Therefore, it is of great interesting to explore the crosstalk and underlying mechanism between miR-135-5p and TGF-β/SMAD signaling pathway in BC metastasis.

In the present study, we revealed that miR-135-5p expression was negatively correlated with BC metastasis and clinical stages, and revealed an inverse relationship between the expression of miR-135-5p and SMAD3 in BC cells and tissues. Importantly, we clarified that miR-135-5p represses TGF-β-mediated EMT and BC metastasis by negatively regulating SMAD3 and TGF-β/SMAD signaling. Collectively, the present study revealed a crosstalk and underlying molecular mechanism between miR-135-5p and SMAD3 in regulating TGF-β-mediated EMT and cell invasion in BC.

## Materials and methods

### Human BC tissues

Sixty-six fresh paired BC and adjacent noncancerous breast tissues were collected from The First People's Hospital of Lianyungang (Lianyungang, China) between May 2015 and September 2018. Among the 66 female patients enrolled in this research: 36 breast cancer patients (mean age, 61.5 years; range, 35-74 years), while another 30 patients with metastatic BC (mean age, 66.6 years; range, 43-82 years). BC patients were diagnosed in accordance with the revised International System for Staging Breast Cancer 21. All BC patients enrolled in this study had signed informed consent and received no radiotherapy or chemotherapy before surgery. Freshly resected BC samples were snap-frozen in liquid nitrogen, and stored at -80 °C for long-term storage. The clinicopathological parameters of the 66 paired tissues are presented in Table 1. This study was approved by the Ethics Committee of The First People's Hospital of Lianyungang.

### Cell culture and cell transfection

The normal human breast epithelial cell line MCF-10A and human BC cell lines MDA-MB-231 and MCF-7 were purchased from the American Type Culture Collection (ATCC). The MCF-7 and MDA-MB-231 cells were cultured in RPMI-1640 medium (Gibco/ThermoFisher Scientific, Waltham, MA, USA) with 10% fetal bovine serum (Hyclone, GE Health Life Sciences). MCF-10A cells were cultured in RPMI-1640 medium with 10% fetal bovine serum and 100 ng/ml cholera toxin (Sigma-Aldrich; Merck KGaA). Cells were maintained at 37 °C in a humidified incubator with 5% CO_2_. The synthetic small interfering RNAs (siRNAs) target SMAD3 (si-SMAD3-1 and si-SMAD3-2) and miR-135-5p mimic/inhibitor/negative controls were obtained from GenePharma (Shanghai, China). Cells were first cultured at 6-well-plates, and were then transfected using Lipofectamine^®^ 2000 (Invitrogen; Thermo Fisher Scientific, Inc., Waltham, MA, USA) when the cells reached 70‑80% confluence, under the guidance of the manufacturer's instructions. BC cells were collected for RT-qPCR verification and further experiments, at 48 h post-transfection. The sequences were: miR-135-5p NC (miR-NC)/si-NC, sense 5'-UUCUCCGAACGUGUCACGU-3', si-SMAD3-1, sense 5'-GCGUGAAUCCCUACCACUA-3', si-SMAD3-2, sense 5'-GCCAUCCAUGACUGUGGAU-3'; miR-135-5p mimics (miR-135-5p), sense 5'-UAUGGCUUUUUAUUCCUAUGUGA-3', and antisense 5'-UCACAUAGGAAUAAAAAGCCAUA-3'; miR-135-5p inhibitor NC (anti-miR-NC), sense 5'-CAGUACUUUUGUGUAGUACAA-3'; miR-135-5p inhibitor (anti-miR-135-5p), sense 5'-UCACAUAGGAAUAAAAAGCCAUA-3'.

### Generation of BC cells stably overexpressing miR-135-5p

To generate stably cell lines overexpressing miR-135-5p in MDA-MB-231 cells, the miR-135-5p or miR-NC segment was synthesized and packaged into pLKO.1-puro-vector (InovoGen Tech. Co.) using the sites of *Eco*RI and *Age*I (Fermentas; Glen Burnie, MD, USA) to generate pLKO.1-puro-miR-NC or pLKO.1-puro-miR-135-5p plasmids. Then, 293 T cells co-transfected with the packaging plasmids (pMD2.G and psPAX2) and the aforementioned plasmids were cultured to product lentivirus. MDA-MB-231 cells were infected with the lentivirus and cultured for 96 h. Finally, MDA-MB-231 cells were selected with puromycin (2 μg/ml; Beijing Solarbio Science & Technology Co., Ltd.,) to achieve stable cell lines. The sequence of miR-135-5p segment was as follows:5'-AGGCCUCGCUGUUCUCUAUGGCUUUUUAUUCCUAUGUGAUUCUACUGCUCACUCAUAUAGGGAUUGGAGCCGUGGCGCACGGCGGGGACA-3'.

### Dual-luciferase reporter assay

Using the online bioinformatic algorithms miRPathDB and TargetScan to predict the targeting relationship between miR-135-5p and SMAD3. The wild-type (WT) and mutated (MUT) 3'-UTR of SMAD3 containing miR-135-5p binding sites (positions 2161-2167) was subcloned into the psiCHECK-2 dual-luciferase reporter vector (Promega Corporation). To determine the effects of miR-135-5p on SMAD3-mediated luciferase activity, MDA-MB-231 and MCF-7 cells were co-transfected with miR-NC or miR-135-5p mimics and luciferase reporter constructs (WT or MUT) using Lipofectamine^®^ 2000. Then, at 48 h post-transfection, BC cells were harvested for the determination of luciferase activity using the Dual-Luciferase^®^ Reporter Assay System (Promega Corporation). Results are presented as relative luciferase activities (firefly/*Renilla*) under the guidance of the manufacturer's instructions 22.

### Reverse transcription-quantitative polymerase chain reaction (RT-qPCR) assay

Total RNA was isolated using TRIzol® reagent (Beyotime Institute of Biotechnology) under the guidance of the manufacturer's protocols. Random primers were employed for cDNA synthesis of SMAD3, and the stem-loop primers were designed to achieve the cDNA of miR-135-5p and U6. cDNA was synthesized according to the reported methods with some modification 8. Subsequently, qPCR was conducted using SYBR-Mix kits (Sigma-Aldrich; Merck KGaA). The qRCR reaction conditions consisted of initial preincubation at 95˚C for 5 min, followed by 45 cycles of amplification (95˚C for 5 sec and 60˚C for 30 sec). Primers are summarized in Table 2. β-actin and U6 were used to normalized SMAD3 and miR-135-5p expression, respectively. RT-qPCR assays were carried out three times, relative mRNA levels were determined using the 2^-ΔΔCq^ method 23.

### Western blot assay

Total protein was isolated for western blot according to the reported methods with some modification 8. The primary antibodies used for western blot analysis were as follows: anti-SMAD3 (1:2,000; cat. no. 9513S; Cell Signaling Technology, Inc.), Vimentin (1:3,000; cat. no. 550513; BD Bioscience), Snail (1:2,000; cat. no. 4719; Cell Signaling Technology, Inc.) and β-actin (1:4,000; cat. no. AC004; ABclonal Bioscience). The secondary antibodies were goat-anti-mouse IgG (1:3,000; cat. no. ab205719; Abcam) and goat-anti-rabbit IgG (1:3,000; cat. no. ab6721; Abcam). The relative protein level was normalized to β-actin. The western blot assays were conducted three times and representative images are presented.

### Transwell assays

The migratory and invasive abilities of BC cells were evaluated by Transwell. For the migration experiments, non-Matrigel-coated 8-*µ*m pore size chambers (Corning Incorporated) were used, and the membrane of chambers treated with Matrigel (200 ng/ml; BD Biosciences) were specially used for the invasion assays. BC cells (6 × 10^4^ cells/200 *µ*l) in RPMI-1640 medium with 1% FBS were placed on the upper chamber, then 700 *µ*l RPMI-1640 medium containing 20% FBS was added to the lower chamber. At 5 h post-seeded with cells in the upper chamber, then BC cells were stimulated with TGF-β1 (5 ng/ml) in a CO_2_ incubator for 24 h. The BC cells on the upper membrane were wiped away carefully. The migrated or invaded cells on the lower chamber were treated with 4% polyoxymethylene, and were then stained with 0.1% crystal violet for 6 h. The cells from 5 random fields were imaged and counted under a light microscope. Transwell assays were repeated three times.

### *In vivo* metastasis assays

Ten female BALB/c nude mice (weight, 18-22g) under 5 weeks old were provided by the Laboratory Animal Center of Nanjing Medical University. Mice were divided into 2 groups (LV-miR-NC, and LV-miR-135-5p; n=5 mice/group). The mice were housed under special pathogen-free conditions (25˚C; 50% humidity; 10 h light/14 h dark cycle). LV-miR-NC and LV-miR-135-5p MDA-MB-231 cells (2.5×10^6^ cells/100 *µ*l PBS/mouse) were intravenously injected into the nude mice. At 8 weeks following tail vein injection, the mice were euthanized, and then the lung tissue samples were obtained and treated with Bouin's fluid for metastatic nodule evaluation. Then, using hematoxylin and eosin (H&E) staining to histologically analyze the micrometastases in lung tissue. Animal experiments were approved by the Ethics Committee of The First People's Hospital of Lianyungang, and the operation procedures were under the guidance of the Care and Use of Laboratory Animals by US National Institutes of Health.

### Hematoxylin and eosin (H&E) staining

The mice lung tissue in *in vivo* metastasis assays were treated with 10% neutral formalin overnight, and then embedded in paraffin. H&E staining was performed under the guidance of reported methods 8. Finally, the lung sections sealed with neutral gum were imaged and examined under an optical microscope (Olympus Corporation).

### Statistical analysis

GraphPad Prism 8.02 software (GraphPad, Inc.) were used for statistical analysis. Data were analyzed and presented as the mean ± standard deviation. Differences between two groups were analyzed by paired or unpaired *t* test (two-tailed). Survival curves were plotted by the Kaplan-Meier method with log-rank test. Spearman's rank correlation analysis was used to evaluate the correlation between the expression of SMAD3 and that of miR‑135-5p. *P* values of < 0.05 were considered statistically significant.

## Results

### miR-135-5p was decreased and inversely correlated with SMAD3 expression in BC tissues

**Figure 1:**
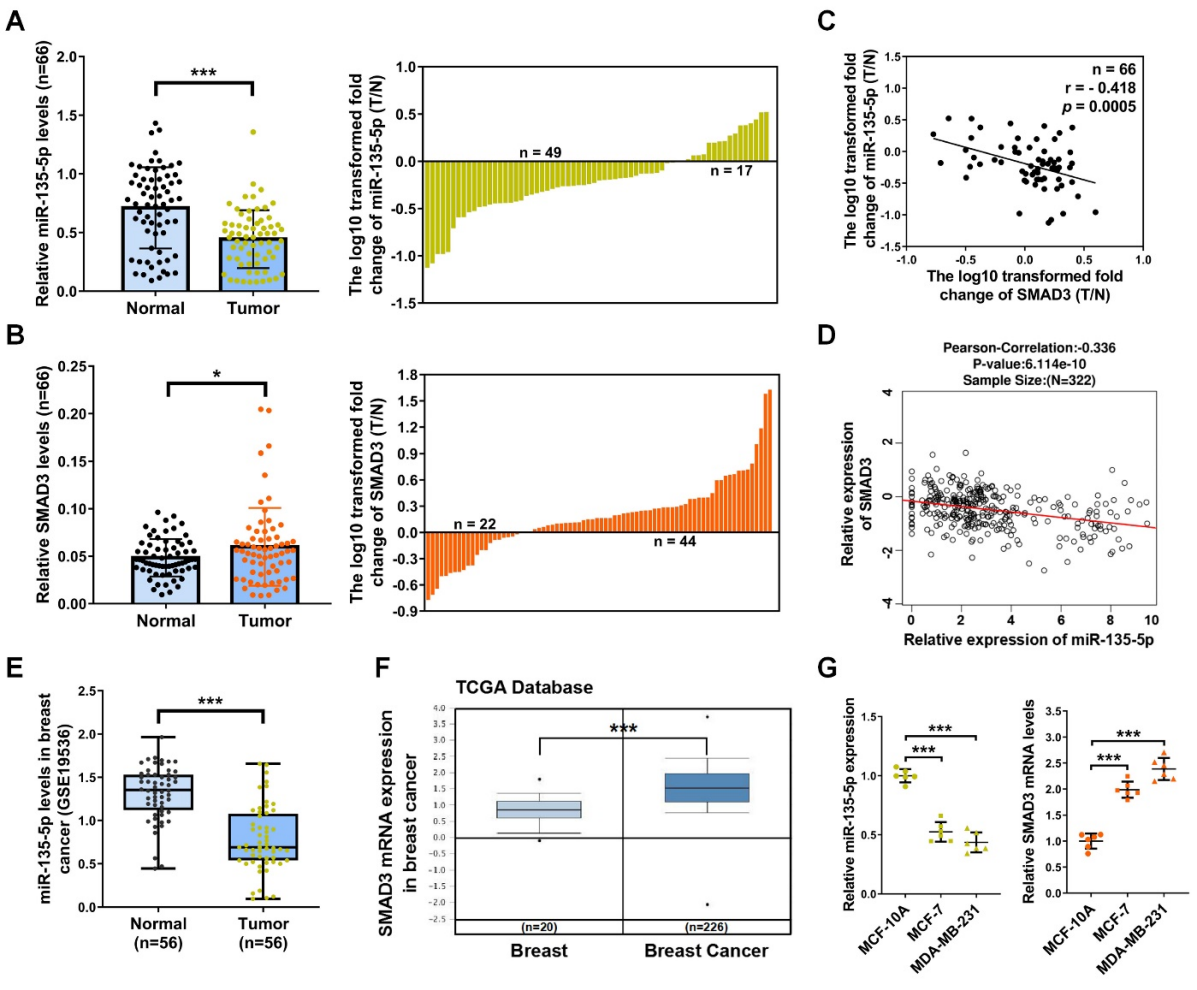
** miR-135-5p expression is decreased and negatively associated with SMAD3 in BC tissues and cell lines**. **(A and B)** The differential expression of miR-135-5p and SMAD3 between 66 paired BC T and N tissues (left panel). The log10 transformed expression levels (T/N) of miR-135-5p and SMAD3 in each sample are shown (right panel). RT-qPCR assays were performed three times. **(C)** Negative association between the mRNA levels of miR-135-5p and SMAD3 in 66 paired BC tissues. X axis shows the log10 transformed mRNA levels (T/N) of miR-135-5p, and Y axIs represents that of SMAD3.** (D)** The negative correlation between miR-135-5p and SMAD3 mRNA levels in BC was verified by LinkedOmics dataset. **(E and F)** miR-135-5p and SMAD3 mRNA levels were repeatedly verified by bioinformatic algorithms Gene Expression Omnibus (GSE19536) and The Cancer Genome Atlas (205398_s_at), respectively. **(G)** RT-qPCR analysis of the mRNA levels of miR-135-5p and SMAD3 in normal human breast epithelial cells and BC cells. U6 and β-actin were used as internal controls. * *P* < 0.05, *** *P* < 0.001.

To investigate the role and relationship of miR-135-5p and SMAD3 in the development and progression of BC, we detected mRNA levels of miR-135-5p and SMAD3 in 66 paired BC and paired adjacent normal tissues. BC tissues showed a significant downregulation in miR-135-5p expression (*P* < 0.001; Fig. 1A) and an obvious upregulation in mRNA expression of SMAD3 (*P* < 0.05; Fig. 1B) when compared with paired adjacent normal tissues. Most important, as illustrated in Table 1, miR-135-5p expression (T/N) was further lower in metastatic BC tissues compared with non-metastatic counterparts (*P* < 0.05). In contrast, SMAD3 mRNA (T/N) were highly expressed in metastatic BC tissues compared with their non-metastatic counterparts (*P* < 0.05). Notably, we observed a markedly inverse correlation between the mRNA expression (T/N) of miR-135-5p and SMAD3 in 66 BC tissues (*P* < 0.001; Fig. 1C), which was further confirmed by the results from the LinkedOmics dataset (http://www.linkedomics.org/login.php) (Fig. 1D). In addition, the database of The Cancer Genome Atlas (TCGA) and the Gene Expression Omnibus were utilized to analyze the expression data of BC tissues. Consistently, GSE19536 dataset indicated that miR-135-5p was decreased in BC tissues (*P* < 0.001; Fig. 1E); TCGA dataset (205398_s_at) verified that the mRNA levels of SMAD3 was increased in BC samples (*P* < 0.001; Fig. 1F). These data strongly supported our findings on miR-135-5p and SMAD3 expression and their relationship in BC. Moreover, RT-qPCR was performed to analyze the mRNA levels of miR-135-5p and SMAD3 in normal human breast epithelial cell line (MCF-10A) and two human BC cell lines (MCF-7 and MDA-MB-231). As shown in Fig. 1G, miR-135-5p expression was significantly decreased in MCF-7 and MDA-MB-231 cells compared with that in MCF-10A cells. On the contrary, BC cells exhibited dramatically increased SMAD3 mRNA levels. In conclusion, the findings reveal a reciprocal expression pattern between miR-135-5p and SMAD3 in BC tissues and cell lines, which indicates a potential role for miR-135-5p in negatively regulating SMAD3 in BC.

### miR-135-5p negatively regulate SMAD3 by directly targeting 3'-UTR of SMAD3

**Figure 2:**
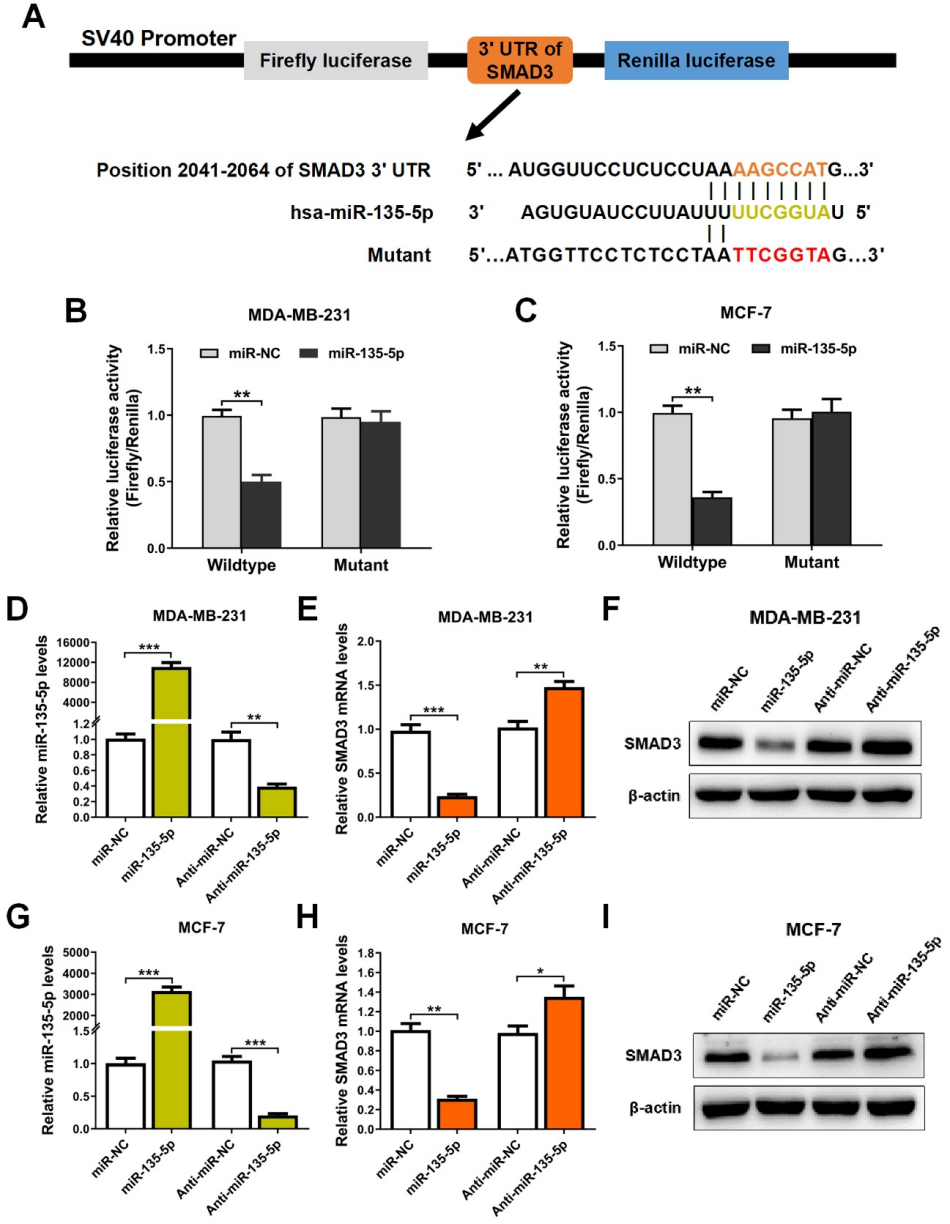
** miR-135-5p negatively regulate SMAD3 by directly targeting 3'-UTR of SMAD3. (A)** Schematic flowchart of the subcloning of potential binding sites between miR-135-5p and SMAD3 3'-UTR in psiCHECK-2 luciferase vector. Prediction binding relationship between SMAD3 and the wild-type/mutant of miR-135-5p. **(B and C)** Results of relative luciferase activities in MDA-MB-231 and MCF-7 cells co-transfected with luciferase reporter constructs (WT or MUT) and miR-135-5p or miR-NC mimics. **(D)** miR-135-5p expression was determined in MDA-MB-231 cells 48 h post-transfection with miR-135-5p or inhibitors. Then, SMAD3 **(E)** mRNA and **(F)** protein levels were analyzed. **(G)** miR-135-5p expression was evaluated in MCF-7 cells, and SMAD3 **(H)** mRNA and **(I)** protein levels were determined. * *P* < 0.05, ** *P* < 0.01, *** *P* < 0.001.

It's well known that miRs suppress target gene expression mainly by binding to the 3′-UTR of mRNAs 24. To examine whether miR-135-5p regulates SMAD3 expression, bioinformatic prediction tools (TargetScan and miRPathDB dataset) were utilized to identify the potential binding sites of miR-135-5p and SMAD3. As illustrated in Fig. 2A, prediction results showed that SMAD3 3'UTR contains a predicted binding sequence for miR-135-5p. In order to verify the prediction results, dual-luciferase reporter analysis was performed in BC cells. The results indicated that miR-135-5p markedly blocked luciferase activity in MDA-MD-231 and MCF-7 cells (*P* < 0.01; Fig. 2B and C), which indicated that miR-135-5p binds to the 3'UTR of SMAD3 and regulate its expression. Importantly, overexpression or knockdown of miR-135-5p significantly decrease or increase the mRNA and protein levels of SMAD3 in MDA-MD-231 (Fig. 2D-F) and MCF-7 (Fig. 2G-I) cells, respectively. Taken together, these data verified our hypothesis that SMAD3 is a direct target gene of miR-135-5p.

### miR-135-5p inhibits TGF-β-mediated EMT and cellular invasion by suppressing TGF-β/SMAD pathway

**Figure 3:**
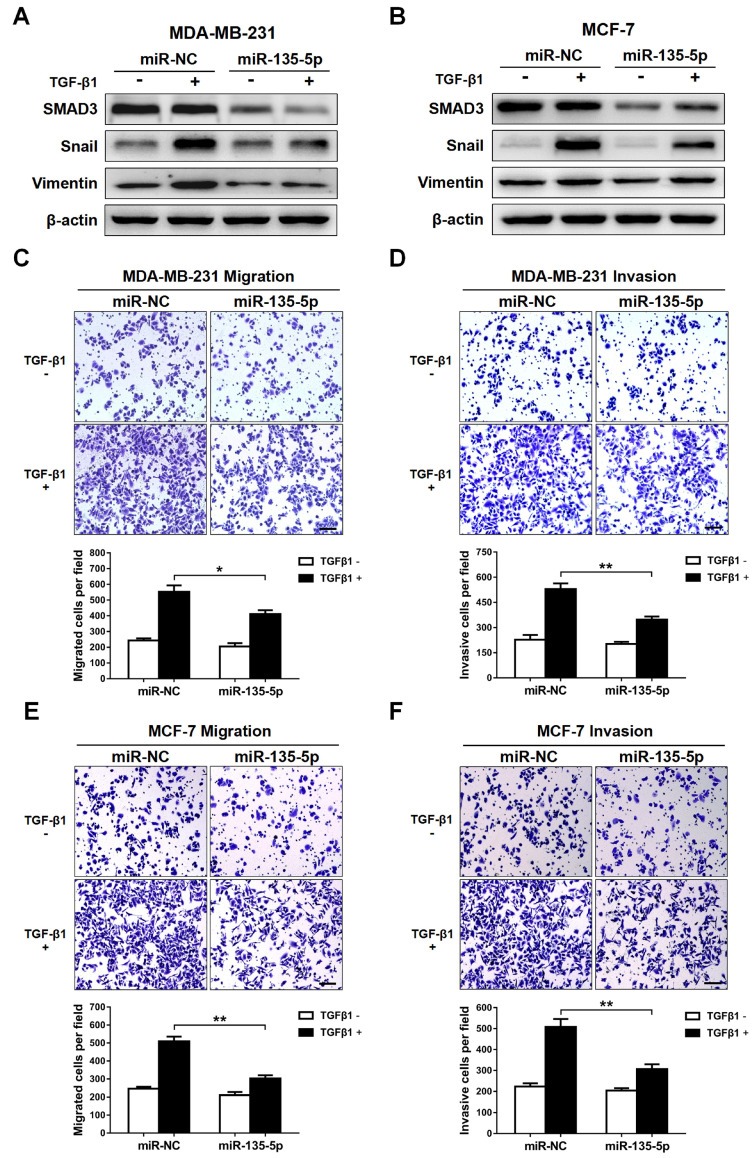
** miR-135-5p inhibits TGF-β-mediated EMT and cellular invasion by suppressing TGF-β/SMAD pathway. (A and B)** Western blot analyze the protein levels of SMAD3, Vimentin and Snail in MDA-MB-231 and MCF-7 cells at 24 h post-transfected with miR-135-5p and negative control, then were stimulated with or without TGF-β1. **(C and D)** Transwell assays quantify the effect of miR-135-5p on the migratory and invasive ability of MDA-MB-231 cells stimulated with or without TGF-β1 for 24 h. Scale bar, 100 *μ*m. **(E and F)** The effect of miR-135-5p on the migratory and invasive ability of MCF-7 cells stimulated with or without TGF-β1 for 24 h was determined by Transwell assays. Scale bar, 100 *μ*m. * *P* < 0.05, ** *P* < 0.01.

Since TGF-β-meidated EMT is a key event during tumor metastasis, western blot and Transwell were conducted to analyze whether miR-135-5p affects TGF-β-mediated EMT and BC cell migration and invasion. The results indicated that ectopic expression of miR-135-5p markedly decreased the protein levels of SMAD3 in MDA-MB-231 and MCF-7 cells (Fig. 3A and B). Furthermore, upon TGF-β1 stimulation, MDA-MB-231 and MCF-7 cells transiently upregulated miR-135-5p expression exhibited reduced protein levels of EMT markers Snail and Vimentin when compared with that of control cells (Fig. 3A and B). Notably, overexpression of miR-135-5p significantly inhibited the migratory and invasive abilities of MDA-MB-231 (*P* < 0.05; Fig. 3C and D) and MCF-7 (*P* < 0.01; Fig. 3E and F) cells stimulated with TGF-β1. In conclusion, these data reveal that miR-135-5p inhibits TGF-β1-mediated EMT and BC cellular migration and invasion by negatively regulating SMAD3 expression and TGF-β/SMAD pathway.

### Knockdown of SMAD3 represses TGF-β-mediated EMT and BC cellular invasion

**Figure 4:**
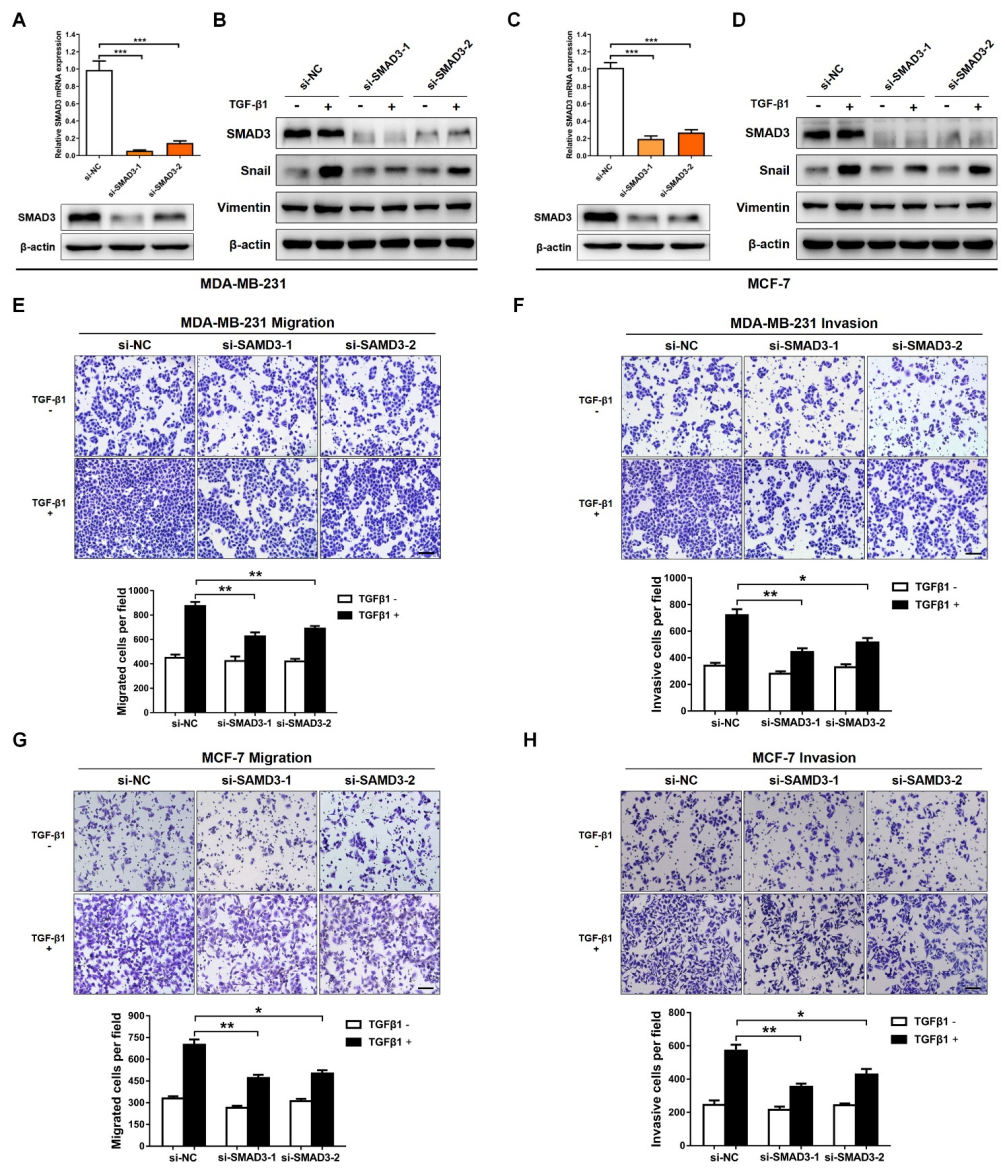
** Knockdown of SMAD3 represses TGF-β-mediated EMT and BC cellular invasion. (A)** Using RT-qPCR and Western blotting to quantify the mRNA and protein levels of SMAD3 in MDA-MB-231 cells transfected with SMAD3-targeted si-RNAs **(B)** Western blot analyze the protein levels of SMAD3, Vimentin and Snail in MDA-MB-231 cells at 24 h post-transfected with SMAD3-targeted si-RNAs, then were stimulated with or without TGF-β1. **(C and D)** Detection of the mRNA levels of SMAD3 and the protein levels of SMAD3, Vimentin and Snail in MCF-7 cells using RT-qRCR and Western blotting, BC cells were treated as above. **(E and F)** The effect of SMAD3 knockdown on the migratory and invasive ability of MDA-MB-231 cells stimulated with or without TGF-β1 was detected by Transwell. Scale bar, 100 *μ*m. **(G and H)** Transwell assays quantify the effect of SMAD3 knockdown on the migratory and invasive ability of MCF-7 cells stimulated with or without TGF-β1. Scale bar, 100 *μ*m. * *P* < 0.05, ** *P* < 0.01.

SMAD3 is a key transcriptional modulator of the TGF-β/SMAD pathway, which plays a critical role in TGF-β-mediated EMT and tumor metastasis 25,26. In order to examine the function of SMAD3, and then evaluate whether SMAD3 knockdown produces a consistent phenotype generated by miR-135-5p overexpression, two pre-designed siRNAs (si-SMAD3-1 and si-SMAD3-2) were synthesized to target SMAD3. As shown in Fig. 4A-D, SMAD3 expression was significantly decreased in MDA-MB-231 and MCF-7 cells transfected with SMAD3 siRNAs. Furthermore, the EMT markers were determined in BC cells stimulated with or without TGF-β1 for 24 h. Results in Fig. 4B and D demonstrated that silence SMAD3 markedly suppressed TGF-β-mediated upregulation of Vimentin and Snail in BC cells. Consistently, the findings of Transwell assays indicated that SMAD3 knockdown inhibited the migratory and invasive abilities of MDA-MB-231 (*P* < 0.05; Fig. 4E and F) and MCF-7 (*P* < 0.05; Fig. 3G and H) cells. Collectively, the results demonstrated that the phenotype generated by miR-135-5p overexpression can be phenocopied by SMAD3 knockdown, which further verified that miR-135-5p suppresses TGF-β-mediated EMT and BC cellular migration and invasion by negatively regulating SMAD3.

### miR-135-5p inhibits BC cell metastasis *in vivo*

**Figure 5:**
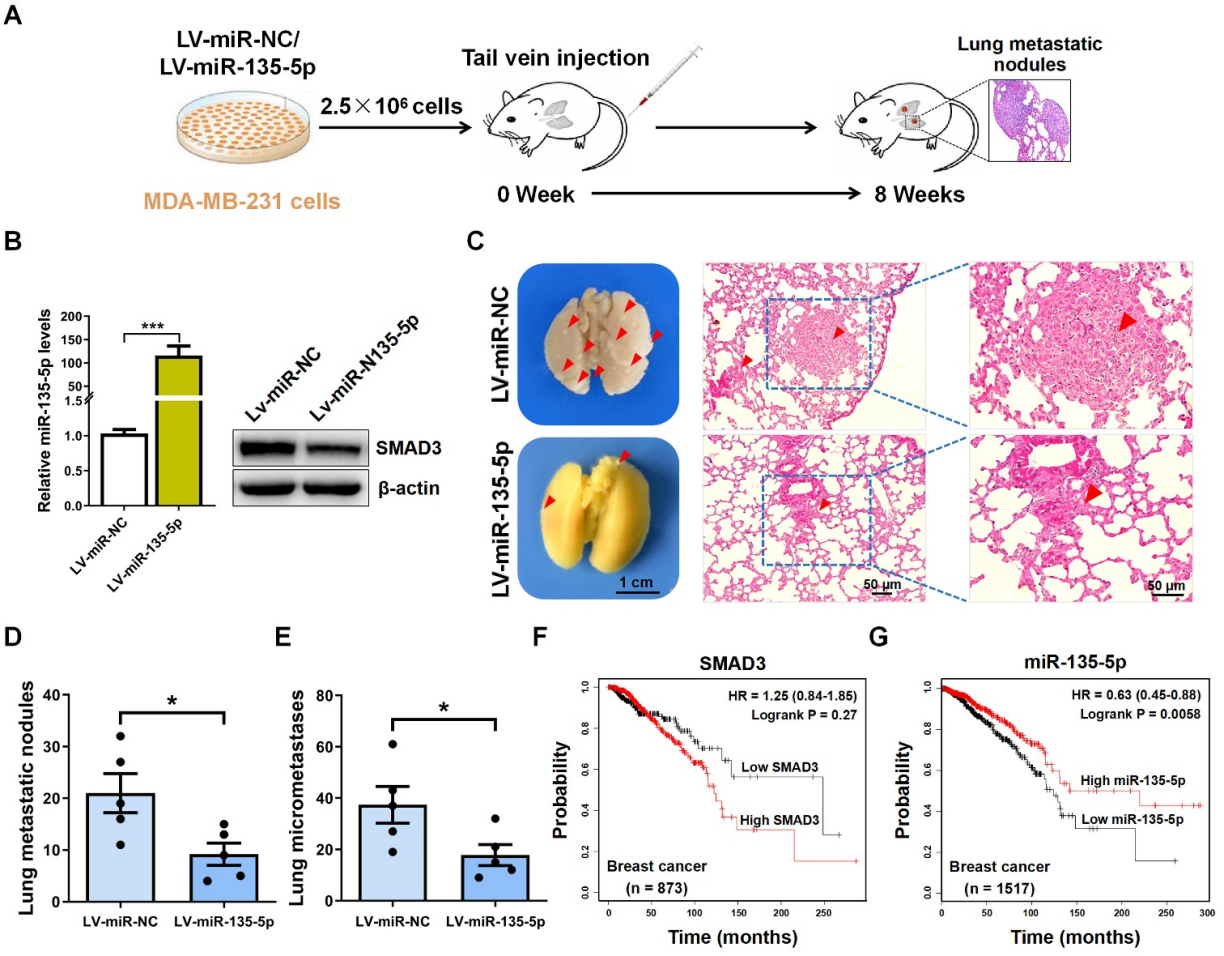
** miR-135-5p inhibits BC cell metastasis *in vivo***. **(A)** Schematic illustration of animal experiments with lung metastases. **(B)** Using RT-qPCR and Western blotting to quantify the expression of miR-135-5p mRNA (left panel) and SMAD3 protein (right panel) in MDA-MB-231 stable cells. **(C)** Representative images showing lung metastatic nodules (left panel) in mice injected with MDA-MB-231 cells (LV-miR-13p-5p or LV-miR-NC). Scale bar, 1 cm. Then, H&E assay evaluated the micrometastases (right panel) in the lung sections; Red arrowheads, metastatic nodules and micrometastases. Scale bar, 50 *μ*m. **(D and E)** Comparison and analysis of the lung metastatic nodules and micrometastases in Fig. 5C. * *P* < 0.05. **(F and G)** Kaplan-Meier plotter dataset was utilized to evaluate the prognostic role of SMAD3 and miR-135-5p in the overall survival of breast cancer patients. * *P* < 0.05.

To explore the role of miR-135-5p in the metastasis of BC cells *in vivo*, MDA-MB-231 cells were intravenously injected into nude mice to establish BC metastasis model (Fig. 5A). A significant upregulated fold-change of miR-135-5p in LV-miR-135-5p MDA-MB-231 cells (*P* < 0.001; Fig. 5B) was detected. Moreover, the protein expression of SMAD3 were markedly decreased in LV-miR-135-5p MDA-MB-231 cells, when it compared to that of LV-miR-NC cells (Fig. 5B). At 8 weeks post-injection of LV-miR-135-5p and LV-miR-NC MDA-MB-231 cells, we euthanized mice and resected lung tissue for analysis of lung metastases and micrometastases. As presented in Fig. 5C and D, mice injected with LV-miR-135-5p MDA-MB-231 cells developed fewer lungs metastatic nodules. Additionally, the results of H&E assay indicated that the number of lung micrometastases was significantly lower in mice injected with LV-miR-miR-135-5p cells as compared to the counterparts (Fig. 5C and E). Furthermore, Kaplan-Meier survival analysis indicated that BC patients with high expression of SMAD3 had shorter overall survival than those with low expression of SMAD3 (Fig. 5F). On the contrary, BC patients with high expression of miR-135-5p had significantly longer overall survival than those with low expression of miR-135-5p (Fig. 5G), which demonstrated that low expression of miR-135-5p may predict a poor prognosis for BC patients 27. In conclusion, these results suggest that the ectopic expression of miR-135-5p inhibits BC cell metastasis* in vivo*.

## Discussion

Many patients when diagnosed with breast cancer is usually in late-stage, with cancer cells invading and metastasizing, which is the main failure factor of clinical treatment for BC patients underwent radiation therapy or chemotherapy 4. TGF-β/SMAD signaling mediated the EMT process has attracted much attention in tumor metastasis research. To date, many miRNAs have been reported to be responsible for EMT and BC metastasis. However, the crosstalk and underlying regulatory mechanism between microRNA and TGF-β/SMAD pathway in EMT and BC metastasis remain elusive. In the present study, we found that the overexpression of miR-135-5p suppresses SMAD3 and TGF-β-mediated EMT and BC metastasis, which indicates that miR-135-5p/SMAD3 axis has vital implications for BC metastasis and progression.

Recent studies have documented that dysregulation of miR-135 is involved in the initiation and development of various cancers 9-11. The present study explored the anti-metastatic role and underlying molecular mechanism of miR-135-5p in BC. Our findings demonstrated that the expression of miR-135-5p was pathologically decreased in BC tissues; more interesting, miR-135-5p expression (T/N) was further downregulated in metastatic BC tissues compared with their non-metastatic counterparts. Subsequently, Gain- and loss-of-function experiments revealed that miR-135-5p obviously suppressed TGF-β-mediated EMT and metastasis of BC cells *in vitro* and *in vivo*. Although clinical data indicated that miR-135-5p may play an anti-tumor role and serve as a prognostic bio-marker in BC; however, the correlation between miR-135-5p expression and BC recurrence and therapy resistance is yet to be evaluated. Consistent with our conclusion, Jiang *et al.* revealed that miR-135-5p was markedly decreased in BC cells and inhibits BC progression by negatively controlling the Wnt/β-catenin pathway 11. In contrast, Yang *et al.* demonstrated that miR-135-5p plays an oncogene role in pancreatic cell adaptation to metabolic stress 12. This contradiction is majorly due to tumor heterogeneity in the microenvironment or target genes.

There are multiple or diverse functions of specific miRNAs, usually clarified as oncogenic stimuli or tumor suppressors, which are mainly dependent on their target genes in human cancers 28-30. In order to predict and verify the direct target genes of miR-135-5p, bioinformatic algorithms and dual-luciferase reporter assay were utilized, and ultimately recognized a novel miR-135-5p-targeted gene, SMAD3, which is a crucial transcriptional modulator of TGF-β/SMAD pathway 31. In this study, we found that the SMAD3 mRNA levels were pathologically upregulated in BC tissues; especially, the mRNA levels of SMAD3 (T/N) were further increased in metastatic BC patients compared with non-metastatic counterparts. Furthermore, we discovered that si-SMAD3 suppressed TGF-β-mediated EMT and BC cellular migration and invasion, which indicated that the knockdown of SMAD3 will produce an effect and phenotype consistent with miR-135-5p overexpression. Zhao *et al.* revealed that TGF-β facilitates breast cancer migration and invasion through Smad3 signaling pathways 32. Most important, for the first time, we revealed the crosstalk between miR-135-5p and SMAD3 in TGF-β-mediated EMT and BC metastasis. However, since TGF-β/SMAD pathway plays a dual role in tumor metastasis and progression. It would be of great interesting to explore whether miR-135-5p regulates SMAD3 to affect cell proliferation of BC cells.

Collectively, for the first time, we demonstrate that miR-135-5p suppresses TGF-β-mediated EMT and BC metastasis by negatively controlling SMAD3 *in vitro* and *in vivo*. Moreover, we further establish an inverse relationship between the expression of miR-135-5p and SMAD3 in clinical BC tissues, which reveal a potential anti-metastatic effect of miR-135-5p in BC progression, and provide a new strategy of miR-135-5p-directed diagnostics and therapeutics for advanced BC.

## Figures and Tables

**Table 1:** Fold change ratio (T/N) of miR-135-5p and SMAD3 mRNA levels between various clinicopathological parameters in 66 paired Breast cancer tissues.

Parameter types	n	miR-135-5p (T/N)	SMAD3 mRNA (T/N)
Age, years			
<60	33	1.211±0.367	1.407±0.122
≥60	33	0.793±0.124	1.515±0.188
*P* value		0.502	0.803
Pathological type			
Non-invasive Carcinoma	46	0.830±0.107	1.422±0.112
Early-invasive Carcinoma	13	0.882±0.208	1.156±0.151
Invasive Carcinoma	7	1.019±0.396	1.067±0.324
*P* value		0.831	0.219
Clinical stage			
I	23	0.922±0.175	1.215±0.154
II	16	0.851±0.208	1.298±0.970
III&IV	27	0.815±0.126	1.454±0.147
*P* value		0.975	0.514
Lymph node metastasis			
No	36	0.997±0.144	1.027±0.115
Yes	30	0.697±0.105	1.982±0.145
*P* value		0.0211	0.047

The fold change ratios (Tumor vs. Normal, T/N) of miR-135-5p and SMAD3 mRNA levels were determined and then analyzed according to various clinicopathologic parameters. Data are shown as mean ± standard error of the mean. Mann-Whitney U test for 2 groups. Kruskal-Wallis test for 3 or more groups.

**Table 2:** Primer sequences for RT and qRT-PCR.

Name	Sequence (5'-3')
RT primers	
U6	CGAGCACAGAATCGCTTCACGAATTTGCGTGTCAT
miR-135-5p	GTCGTATCCAGTGCAGGGTCCGAGGTATTCGCACTGGATACGACTCACATAG
qRT-PCR primers	
U6	F: CGAGCACAGAATCGCTTCA;
	R: CTCGCTTCGGCAGCACATAT
miR-135-5p	F: CAGTGCAGGGTCCGAGGTAT;
	R: CGTCGTATGGCTTTTTATTCC
SMAD3	F: CCATCTCCTACTACGAGCTGAA;
	R: CACTGCTGCATTCCTGTTGAC
β-actin	F: CATGTACGTTGCTATCCAGGC;
	R: CTCCTTAATGTCACGCACGAT

F: forward; R: reverse.
